# Supplementary material for: The genome sequence of dewberry, Rubus caesius L. (Rosales: Rosaceae)
Source: Wellcome Open Res. 2026 Sep 18;11:705. [Version 1] doi: 10.12688/wellcomeopenres.27853.1 (PMC13662391; doi:10.12688/wellcomeopenres.27853.1)
Supplement: Peer review history

# Reviewer report: Reviewer response for version 1

**DOI:** 10.21956/wellcomeopenres.30650.r171097

**Published:** 2026-10-13

**Authors:** Marco Todesco

**Affiliations:** 1 University of British Columbia, Vancouver, Canada

This genome note reports the genome assembly of the allotetraploid European dewberry ( *Rubus caesius*). Despite the more complex ploidy, the assembly can resolve very well what are likely the two sets of 7 ancestral chromosomes, with what appears to be almost no uncertainty based on the Hi-C heatmaps.

 Unfortunately, it looks like no gene annotation was produced in this case, which would have been quite interesting for understanding how the two chromosome complements have evolved since the original hybridisation event that produced *R. caesius*.

 Nonetheless, this is a great resource that will undoubtedly be of great use in resolving taxonomic relationships between wild Rubus species. I only regret never having tasted European dewberry, despite having lived in Italy and Germany for decades.

Are the datasets clearly presented in a usable and accessible format, and the assembly and annotation available in an appropriate subject-specific repository?

Yes

Are sufficient details of the sequencing and extraction, software used, and materials provided to allow replication by others?

Yes

Are the rationale for sequencing the genome and the species significance clearly described?

Yes

Are the protocols appropriate and is the work technically sound?

Yes

Reviewer Expertise:

plant genetics, adpatation, genomics

I confirm that I have read this submission and believe that I have an appropriate level of expertise to confirm that it is of an acceptable scientific standard.

# Reviewer report: Reviewer response for version 1

**DOI:** 10.21956/wellcomeopenres.30650.r171098

**Published:** 2026-10-13

**Authors:** Rohit Joshi

**Affiliations:** 1 CSIR-Institute of Himalayan Bioresource Technology, Palampur, Himachal Pradesh, India

The manuscript looks good in its present shape.

 The dewberry genome will add new knowledge to the existing one. 

 The description of morphological characteristics is clear.

 Provide distinction between the genome-size estimate for one basic chromosome set (337.59 Mb) and the ploidy-adjusted haploid estimate (675.18 Mb)

 Check reference formatting.

 Use consistent British or American English spelling

Are the datasets clearly presented in a usable and accessible format, and the assembly and annotation available in an appropriate subject-specific repository?

Yes

Are sufficient details of the sequencing and extraction, software used, and materials provided to allow replication by others?

Yes

Are the rationale for sequencing the genome and the species significance clearly described?

Yes

Are the protocols appropriate and is the work technically sound?

Yes

Reviewer Expertise:

Plant Biotechnology

I confirm that I have read this submission and believe that I have an appropriate level of expertise to confirm that it is of an acceptable scientific standard.

# Reviewer report: Reviewer response for version 1

**Published:** 2026-09-18

**Authors:** Automated Benchmarking

**Affiliations:** 1 Wellcome Sanger Institute, Hinxton, UK

As this article is a Genome Note, Automated Benchmarking has been used to determine whether the reported assembly passes four defined checks established by the Earth BioGenome Project: the raw and assembled data are publicly available, the Earth BioGenome Project three-part metric is met, the k-mer completeness exceeds 90%, and the BUSCO score passes the stated threshold. For Rubus caesius (drRubCaes1), all four checks passed. Data available: pass. ENA study PRJEB74720, raw-read accessions ERR12875186 and ERR12893042, and assembly accessions GCA_964235055.1 and GCA_964235075.1 are public. Earth BioGenome Project three-part metric: pass. Haplotype 1 is reported as 6.C.Q61, meeting the 6.C.Q40 reference standard. k-mer completeness: pass. The combined haplotype assemblies have 98.63% k-mer completeness, exceeding the Earth BioGenome Project target of 90%. BUSCO: pass. Haplotype 1, which is proposed as the reference assembly for this species, has 98.6% complete BUSCOs (8.1% single-copy and 90.5% duplicated). BUSCO software version: 5.5.0; lineage dataset: eudicots_odb10. It meets the plant-specific target of greater than 90% complete BUSCOs. This decision uses values reported in the Genome Note and public accession records.

Reviewer Expertise:

NA

I confirm that I have read this submission and believe that I have an appropriate level of expertise to confirm that it is of an acceptable scientific standard.
